# Outbreak of Cutaneous Leishmaniasis among military personnel in French Guiana, 2020: Clinical, phylogenetic, individual and environmental aspects

**DOI:** 10.1371/journal.pntd.0009938

**Published:** 2021-11-19

**Authors:** Kim Henry, Aurélie Mayet, Miguel Hernandez, Guillaume Frechard, Pierre-Antoine Blanc, Marion Schmitt, Nathalie André, Jean-Marie Loreau, Marine Ginouves, Ghislaine Prévot, Pierre Couppié, Magalie Demar, Romain Blaizot

**Affiliations:** 1 Laboratory of Parasitology-Mycology, Centre Hospitalier de Cayenne, Cayenne, French Guiana; 2 French Military Health Service—Armed Forces Epidemiology and Public Health Center, Marseille, France; 3 Aix Marseille University, INSERM, IRD, SESSTIM, Economic and Social Sciences of Health and Medical Information Processing, Marseille, France; 4 National Reference Center for Leishmaniasis, associate laboratory, Cayenne, French Guiana; 5 French Military Health Service—Kourou Medical Center, Kourou, French Guiana; 6 French Military Health Service—Cayenne Medical Center, Cayenne, French Guiana; 7 French Military Health Service—Inter Army Directorate of the Armed Forces Health Service, Cayenne, French Guiana; 8 UMR 1019 Tropical Biomes and Immuno-Physiopathology, University of French Guiana, Cayenne, French Guiana; 9 Univ. Lille, CNRS, Inserm, CHU Lille, Institut Pasteur de Lille, U1019—UMR 9017—CIIL—Center for Infection and Immunity of Lille, Lille, France; 10 Dermatology Department, Centre Hospitalier de Cayenne, Cayenne, French Guiana; Centro de Pesquisa Gonçalo Moniz-FIOCRUZ/BA, BRAZIL

## Abstract

**Background:**

Cutaneous Leishmaniasis (CL) is endemic in French Guiana but cases are usually sporadic. An outbreak signal was issued on May 15^th^ 2020 with 15 suspected cases after a military training course in the rainforest. An outbreak investigation was carried out.

**Methodology/Principal findings:**

Thirty cases were confirmed. *Leishmania guyanensis* was the most frequent species (90%). The most frequent presentation was ulcerative (90%). Lesions on the face and hands were frequent (40% each). Eight cases (26%) presented a poor outcome after treatment with pentamidine and required a second line with amphotericin B. Three of them required further treatments with meglumine antimoniate or miltefosine. Two spots within the training area were deemed as likely sites of contamination, due to illegal logging. The isolated *Leishmania* strains did not form a separate cluster. Participation in Week 13 of year 2020 was associated with infection (OR = 4.59 [1.10–19.83]; p = 0.016) while undergoing only the “Fighting” exercise was protective (OR = 0.1 [0–0.74]; p = 0.021). There was no association between infection and other risk factors at the individual level. The attack rate of Regiment B (14/105 = 13.3%) was significantly higher (OR = 4.22 [1.84–9.53], p = 0.0001) compared to Regiment A (16/507 = 3.2%). The attack rate during this training course (30/858 = 3.5%) was significantly higher (OR 2.29 [1.28–4.13]; p = 0.002) than for other missions in French Guiana during the same period (22/1427 = 1.5%).

**Conclusions:**

This outbreak could be explained by a combination of factors: climatic conditions around week 13, at-risk activities including night trainings, absence of impregnation, a lesser experience of rainforest duties in Regiment B and illegal logging attracting sandflies on military training grounds.

## Author summary

Cutaneous Leishmaniasis is caused by parasites of the *Leishmania* genus and infects humans after a sandfly bite. Outbreaks are rare and hard to investigate in isolated tropical areas. In this study, the authors explored the different possible origins of an outbreak of cutaneous leishmaniasis among soldiers training in the rainforest of French Guiana. The outbreak occurred in March 2020. Concerning the symptoms, several patients presented resistant infections and multiple lines of treatment, raising the issue of resistant *Leishmania* strains. The different strains isolated during the outbreak were not genetically closed, as far as routine PCR techniques would indicate. The authors looked for individual behaviours exposing soldiers to sandfly bites but none was significantly associated with infection. The authors found two spots in the military training areas where illegal logging probably increased the density of sandflies and put service members at risk. The 13^th^ week of 2020 was associated to a higher risk of infection due to climatic conditions. This study shows how interactions between humans and the rainforest can increase the risk of parasitic outbreaks.

## Introduction

Cutaneous Leishmaniasis (CL) is a Neglected Tropical Disease (NTD) affecting 91 countries throughout the world. Three quarters of all cases are reported in the Eastern Mediterranean Region and 18% in the Americas region (46 265 cases/year) [1]. More than 26000 cases are reported each year in Brazil [2]. In French Guiana, between 200 and 300 cases are reported annually [3,4]. In this French territory, five *Leishmania* species are reported: *Leishmania guyanensis*, *L*. *braziliensis*, *L*. *naiffi*, *L*. *lainsoni*, and *L*. *amazonensis* [3,5,6]. *L*. *guyanensis* is the most frequent species and usually represents more than 80% of cases each year [3]. This species is transmitted by the female phlebotomine sandfly *Nyssomyia umbratilis*, which has a sylvatic cycle [7]. *Choloepus* d*idactylus*, or two-toed sloth, is the main reservoir for *L*. *guyanensis* in French Guiana [4,8]. *L*. *braziliensis* is the second most frequent species (10%] and is characterized by a high risk of mucosal infection [3].

The diagnosis of CL relies on clinical signs and microbiological confirmation. Smear is commonly used in French Guiana but does not provide species identification. As treatment differs between *L*. *guyanensis* and *L*. *braziliensis*, this identification is paramount for proper clinical care [3] Species identification is usually performed through Matrix Assisted Laser Desorption Ionization—Time of Flight (MALDI-TOF) or Polymerase Chain Reaction (PCR) followed by DNA Sanger Sequencing obtained from parasites cultured from skin biopsy or impregnate on cotton swabs [9]. Most contaminations occur during professional forest activities in farmers, gold miners and soldiers [3]. Most cases are sporadic and seen between November and May [3,6], though explanations for this apparent seasonality remain controversial. A decrease in rainfall during the dry season has been linked to an increase in CL cases two months later, possibly due to frequent forest activities during the dry months.[10]

Only three CL outbreaks have been described in French Guiana so far. Two occurred in military personnel in 1998 [11] and 2002 [12] and a third one in scientists infected with *L*. *braziliensis* during a forest trip [13]. This latter study was the only one involving phylogenetic analysis. Indeed, strains are rarely isolated during outbreaks due to the technical difficulties of collecting samples on the field. Besides, these outbreaks were limited in size and environmental on-field investigation was not performed.

We report here a large outbreak of Cutaneous Leishmaniasis occurring in military personnel in French Guiana. We discuss clinical presentations and treatment response, microbiological characteristics and factors associated with contamination such as environmental triggers or human behaviors.

## Methods

### Ethics statement

This study (under the name CEFELEISH) was authorized by the Strasbourg *Comité de Protection des Personnes* (CPP) Est IV (1 Place de l’Hôpital—Bât PGIL 1 er étage– 67091 Strasbourg, France), with the identification number 2020-A02327-32. All patients gave their vocal assent to be included in the study. All symptomatic cases had provided previous written assent for routine clinical data collection while being treated at the Cayenne Hospital Center. No medical data were recorded from the controls and written assent was therefore unnecessary, according to the current French law.

On May 15^th^ 2020, the Cayenne Inter-Army Medical Center referred 15 suspected cases of CL to the Dermatology Department of the Cayenne Hospital. In the following weeks, 36 others suspected cases of CL in service members were also referred by the Cayenne and Kourou military medical centers. Most patients belonged to two military units which will be named A and B in the present article. Medical histories suggested a common source of contamination during military training in the CEFE (Training Center in Equatorial Forest).

The CEFE is a French military training ground set in deep rainforest on the commune of Regina, 80 km away from the nearest town (Saint-Georges). This center welcomes all kind of military personnel and provides them with specialized training for survival and fighting in the Guyanese rainforest. However, personnel from units A and B represent most of trainees, due to their role in defending the territory of French Guiana and fighting illegal gold mining. Many different trainings are offered in the CEFE. At the time of this outbreak, the two most frequent courses were entitled Fighting (*Combat*) and Survival (*Survie*). The former lasts between one and two weeks while the latter usually lasts a week. A longer course (*Jaguar*) is sometimes also offered. Some of the instructors are permanently stationed in the CEFE.

This outbreak investigation was divided in four parts.

### Clinical study

In order to investigate the clinical pattern of this outbreak, we gathered information on all cases of military personnel with suspected CL seen during the first semester of 2020 in the Cayenne Hospital or the Inter-Armies Medical Centers of Cayenne and Kourou. We excluded military personnel who did not undertake a CEFE course or did so before January 1st 2020 or after June 30th 2020. We also excluded service members with cutaneous leishmaniasis if the symptoms began more than 3 months after their CEFE course AND if they had performed other forest missions in the meanwhile, as the relationship between infection and the CEFE was deemed unlikely. A confirmed case of CL was defined as compatible clinical presentation AND at least one positive laboratory test (smear, culture on skin biopsy, MALDI-TOF on biopsy culture, PCR on cotton swab) OR complete response after empirical therapy with pentamidine.

Patients were always seen one month after treatment: good response was then defined as decrease in lesions size by at least 50%, with no new lesions. Patients were then seen after a three-months follow-up: good response was then defined as healing of all lesions, with no new lesion. These clinical criteria are those usually used in our Dermatology Department [14].

### Phylogenetic comparisons

A phylogenetic comparison was made between the different strains isolated during the outbreak. PCR on cotton swabs was performed according to previously published protocols [9]. QIAamp DNA Mini Kit (QIAGEN, Hilden, Germany), was used to extract the parasite genomic DNA from cotton swabs, according to the manufacturer’s instructions. The Hsp70 gene then subsequently amplified by PCR using primers Hsp70senM (5’-GACGGTGCCTGCCTACTTCAA-3’) and Hsp70ant (5’CCGCCCATGCTCTGGTACATC 3’). PCR was performed using the following mixture: 0.112μM primers, 10 μL *5x HOT FIREPol Blend Master Mix* (Solis BioDyne, Tartu, Estonia) and 10 μL of DNA template, for a final volume of 50 μL. Reaction cycles included initial denaturation for 14 min at 95°C, then 40 cycles of 30s at 95°C; followed by cycles of 45s at 60°C and 1.5 min at 72°C; and a final 5 min extension step at 72°C. Amplified products (»1500 bp) were visualized by electrophoresis on 1% agarose gels, and were sent to Genoscreen (Lille, France) for DNA sequencing.

Chromatograms obtained from DNA Hsp70 sequence were visualized using Chromas software, (version 2.6.5, Technelysium Pty. Ltd., Tewantin, Queensland, Australia), corrections were made by visualization. Consensus Hsp70 sequences were obtained using Bioedit version 7.2. Consensus sequences were then subjected to BLASTn on GenBank (NCBI web site) to look for similarity with *Leishmania* reference sequences and achieve correct species identification.

Consensus sequences were aligned by using T-coffee software (http://tcoffee.crg.cat/apps/tcoffee/index.html). A phylogenetic tree was then built for each gene Using MEGA software 7.0.26 (Penn State University, PA, USA). Distances from nucleotide sequences were estimated with the Kimura-2 parameter model, and trees were built with the maximum likelihood (ML) method and bootstrap resembling was used across 1000 replicates. Phylogenetic trees were built using all confirmed cases of CL with positive PCR and available clinical data. Comparison was made by using reference strains from the literature and 25 cases of CL observed in civilians throughout French Guiana during the study period.

### Epidemiological study

A case-control study was performed to evaluate behavioural patterns associated with the occurrence of CL infection. Cases were patients included in the clinical study who also agreed to answer supplementary questions. Controls were randomly selected service members who undertook a CEFE course between January 1^st^ 2020 and 30^th^ June 2020 without symptomatic CL infection and agreed to be included. Cases and controls were called and interviewed by phone by the same investigator (KH).

### Environmental investigation

An on-field investigation was performed in August 2020 on the CEFE site. A team was dispatched to review the military gear used during CEFE courses, the different training spots and places of encampment. The team notably looked for spots where sandflies could be drawn into contact with humans, like streams, recently cleared or forested areas, encampments … We also looked for climatic factors which could explain the increased number of cases in this period of 2020.

### Statistics

The relationship between individual risk factors and *Leishmania* infection was analyzed using Fisher’s exact test or χ2 test, as appropriate. Statistical analyses were performed using Stata Software (StataCorp, College Station, USA) with a significance bilateral threshold of 0.05.

## Results

### Clinical study

**Fig 1:**
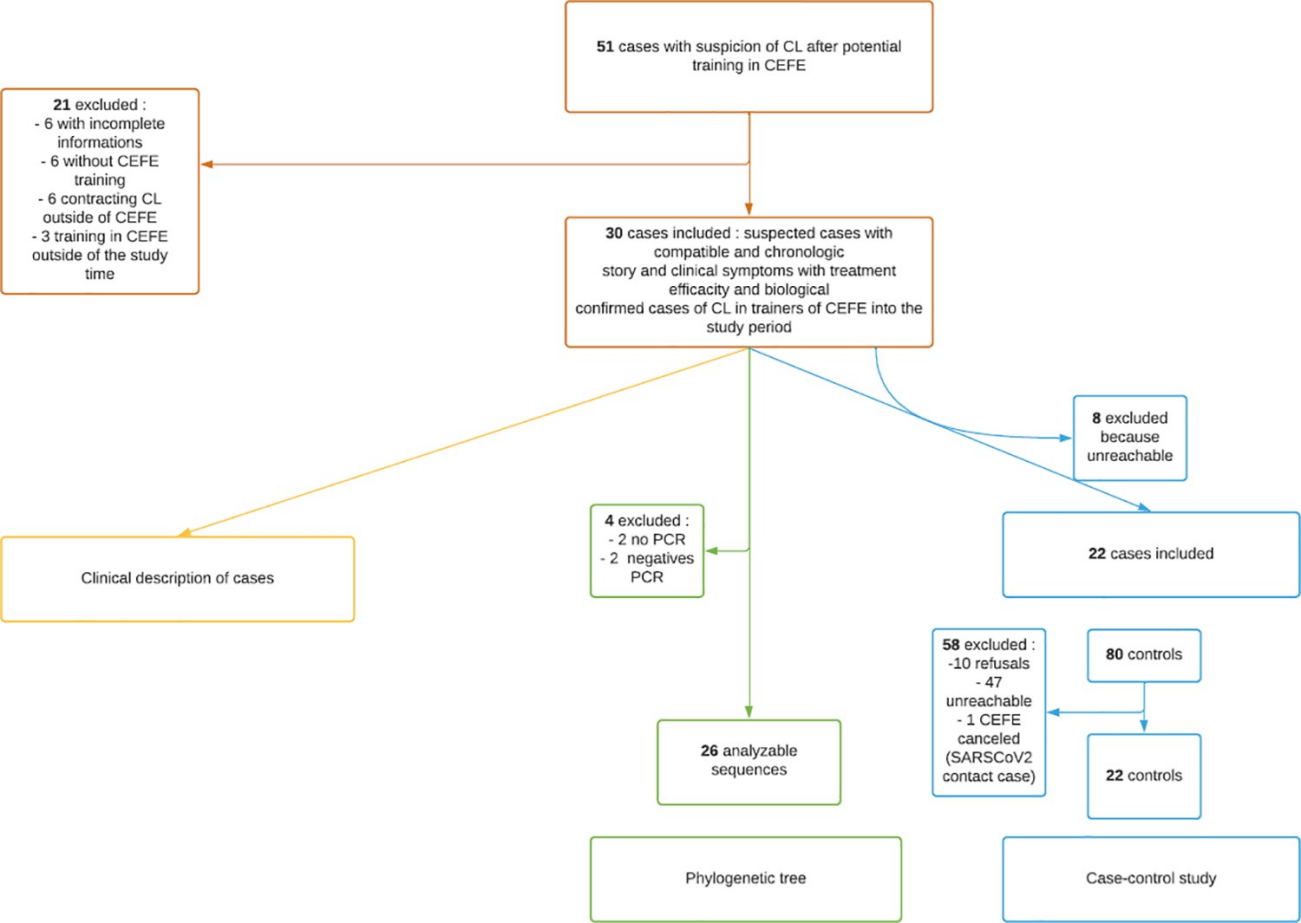
Flow chart of patients respectively included in the clinical description of cases, phylogenetic analysis and case-control study, CEFE outbreak, French Guiana, 2020.

Between January 1^st^ 2020 and June 30^th^ 2020, 51 service members were referred to our Department or to the Inter-Army Medical Center of Kourou with suspected CL. Twenty-one were excluded (**Fig 1**). In total, we included 30 soldiers with confirmed CL who had undertaken a CEFE course during the first semester of 2020. Among them, 22 were included in the case-control study, 26 in the phylogenetic study. Inclusions the three arms of the study are summed up in this flow-chart (**Fig 1)**.

The clinical characteristics of the thirty included cases are presented in **Table 1**.

**Table 1:** General characteristics of confirmed cases of Cutaneous Leishmaniasis, CEFE outbreak, French Guiana, 2020 (n = 30).

	Number of patients, (%)
**Mean age (year)**	32
**Gender**: male	30 (100%)
**Military unit**	
A	16 (53.3%)
B	14 (46.7%)
**CEFE period (2020 calendar weeks)**	
Instructor (always present)	1 (3.3%)
Week 4 to Week 12	1 (3.3%)
Weeks 6–7	1 (3.3%)
Week 13–14	20 (66.7%)
Week 16–17	4 (13.3%)
Week 17–20	2 (6.7%)
Week 19–20	1 (3.3%)
**CEFE activity**	
Fighting	24 (80.0)
Other trainees	4 (13.3%)
Rainforest specialist	2 (6.67%)
International training course « Jaguar »	2 (6.67%)
Instructor	2 (6.67%)
**Previous history of CL**	0
**Lesion location**	
Upper limbs	19 (63.3%)
Hands	12 (40%)
Lower limbs	13 (43.3%)
Trunk	7 (23.3%)
Face	12 (40%)
Neck	6 (20%)
Scalp	2 (6.7%)
**Lesion type**	
Ulceration	27 (90%)
Nodule	6 (20%)
Papule	5 (16.7%)
Other	1 (3.3%)
**Mean number of lesions (1–30)**	4.33
**Median number of lesions**	3 (1–6)
**Mucosal lesion**	0
**Lymphadenopathy**	4 (13.3%)
**Lymphangitis**	1 (3.3%)
**Laboratory tests**	
Positive smear	16 (53.3%)
Positive culture	11 (36.7%)
Including MALDI-TOF identification	11 (36.7%)
Positive PCR	26 (86.7%)
**Parasite species:**	
*Leishmania guyanensis*	27 (90%)
*Leishmania* spp	1 (3.3%)
Unknown (no PCR or negative PCR)	2 (6.6%)
**Treatment: 1st line (n = 30)**	
Pentamidine	30 (100%)
**Response at 1 month (n = 30)**	
Good response	15 (50%)
Bad response/failure	10 (33.3%)
Lost to follow-up	5 (16.7%)
**Treatment: 2**^**nd**^ **line (n = 8)**	8 (26.7%)
Pentamidine (2^nd^ injection)	2 (25%)
Amphotericin B	6 (75%)
**Response at 3 months (n = 8)**	8
Good response	2 (25%)
Bad response	3 (37.5%)
Lost to follow-up	3 (37.5%)

All of them were men, with a median age of 31 (IQR [26–51]). Regarding medical histories and outcomes, there was no recorded comorbidity such as HIV infection, diabetes, renal failure, overweight or immunosuppressive therapy. The most frequent location of lesions was upper limbs in 19 patients (63.33%), followed by lower limbs (13, 43.3%). The face and hands were frequently involved (12 patients each, 40%). The most frequent type of lesion was ulceration (27 patients, 90%). We recorded no mucosal involvement.

Concerning laboratory tests, 26 patients (86.7%) had a positive PCR on cotton swabs, 16 (53.3%) had a positive smear, 11 (36.7%) had a positive culture which allowed MALDI-TOF for species identification. The infecting *Leishmania* species was identified for 90% of cases (27 patients) and was always *Leishmania guyanensis*. Among these 27 patients, species identification was allowed by PCR and confirmed by MALDI-TOF in 9 of them, by PCR only in 17 patients (56.7%), and by MALDI-TOF only for two patients. In the Cayenne Hospital Centre, PCR on cotton swabs and parasitological cultures on skin biopsy are performed in parallel. MALDI-TOF on culture is impossible when cultures are contaminated by bacteria or fungi, but sometimes yields a species identification while PCR in the same patient is negative.

All thirty patients received a first-line treatment with pentamidine isethionate. Due to different protocols in the Military Center and the Cayenne Hospital, 17 of them (56.7%) received three intravenous injections of 4mg/kg/d [15] while 12 patients (40%) received one intramuscular injection of 7mg/kg/d [3]. One was treated in mainland France with pentamidine but it is not known which protocol was used. Half of cases (15 patients) presented a complete response after this first line of treatment, regardless of the regimen used (one or three injection). Five patients (16.7%) were lost to follow-up.

One third of cases (10) did not present a good response to this first line of treatment, and eight patients (26.7%) received a second line with amphotericin B (4mg/kg/d for five days) [14] while the two others were lost to follow-up. Among these 10 patients who presented a bad outcome after the first scheme, five had received three IV injections and five had received one IM injection. There was no significant difference in bad outcome between these two groups (OR 0.58 [0.09–3.62], p = 0.49).

**Fig 2:**
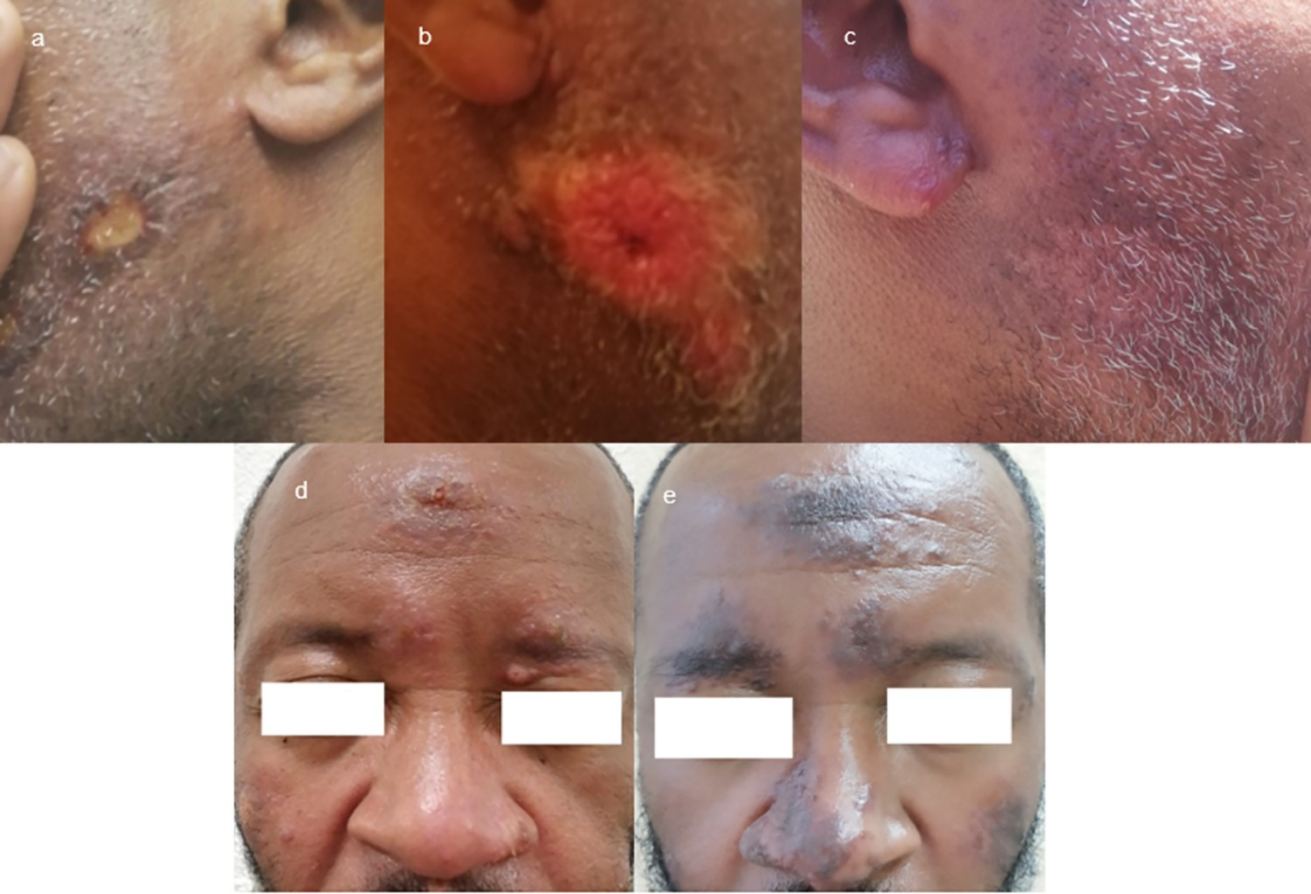
**Clinical evolution of patient 1, CEFE outbreak, French Guiana, 2020:** initial lesion of the left cheek (**2a**), which improved after pentamidine; new lesion on the opposite cheek (**2b**) improved after amphotericin B **(2c**); new relapse occurred on the right ear and the forehead (**2d**); after another unsuccessful amphotericin B course and three weeks of meglumine antimoniate (75mg/kg/d), improvement was seen on the face (**2e**) but disseminated new lesions appeared. Healing was obtained after one month of oral miltefosine (50mg tid).

**Fig 3:**
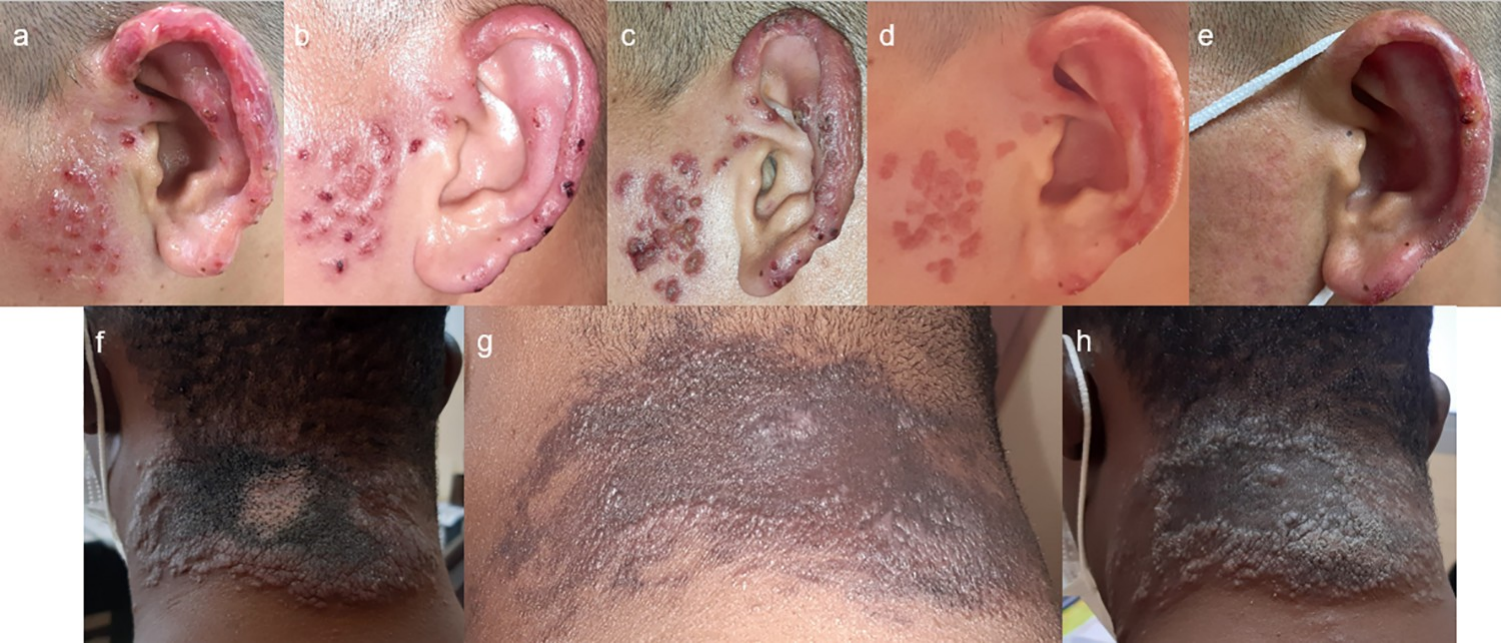
**Clinical evolution of patient 2 and 3, CEFE outbreak, French Guiana, 2020:** patient 2 presented an ulceration on the ear and papules of the adjacent cheek (**3a**); improvement after pentamidine (**3b**) followed by relapses (**3c**), requiring a course of amphotericin B, then a second scheme in association with miltefosine (**3d**), with partial response (**3e**) followed by a relapse. The patient was then treated with 3 weeks of meglumine antimoniate. Patient 3 presented a similar history with a pseudo-verrucous plaque of the neck (**3f**), which first improved (**3g**), and then relapsed (**3h**) motivating a further line of treatment with amphotericin B, amphotericin B and miltefosine then finally meglumine antimoniate.

Among the eight patients who received a second line of treatment, three were lost to follow-up, two presented a good response, and three were not healed and needed a third line of treatment. The clinical history of these three patients is summed up in **Figs 2** and **3**.

**Fig 4:**
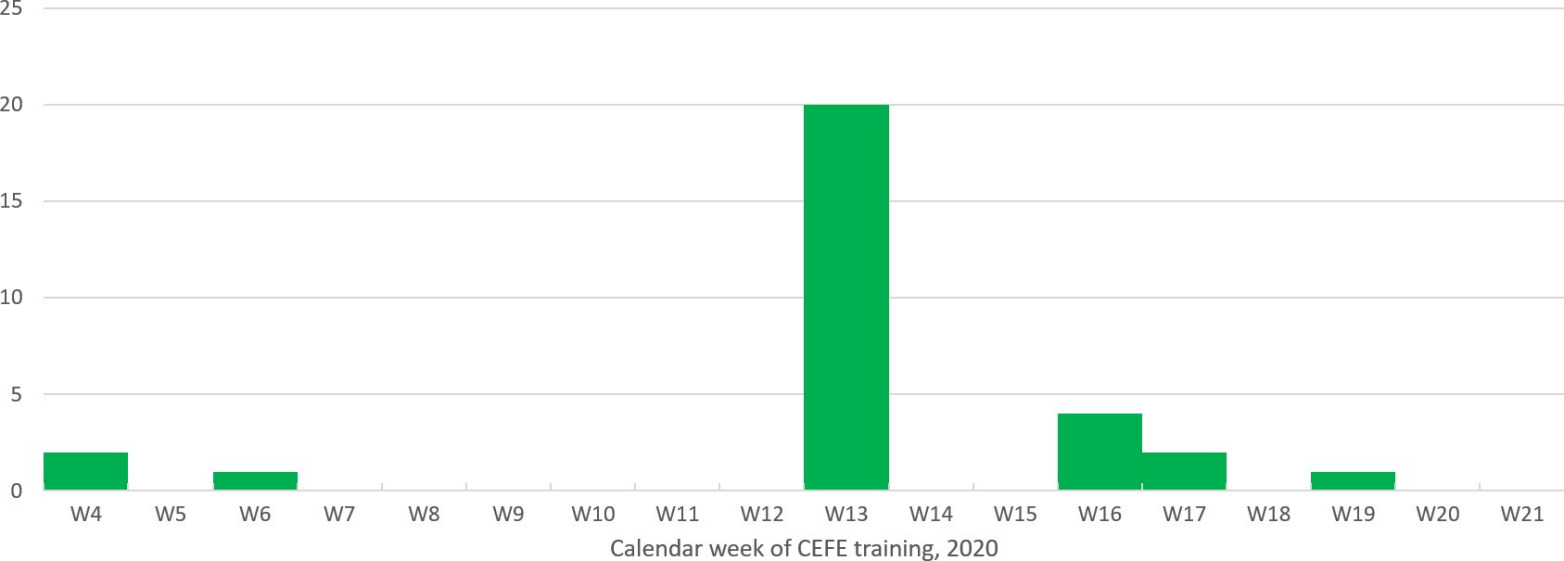
Number of confirmed cases of cutaneous leishmaniasis for each calendar week between February and June 2020, CEFE training site, French Guiana, 2020.

The temporal repartition of confirmed cases is presented in **Fig 4** with the likely time of contamination corresponding to the week of CEFE training for each individual. A peak was observed during week 13 of the calendar year.

### Phylogenetic comparisons

**Fig 5:**
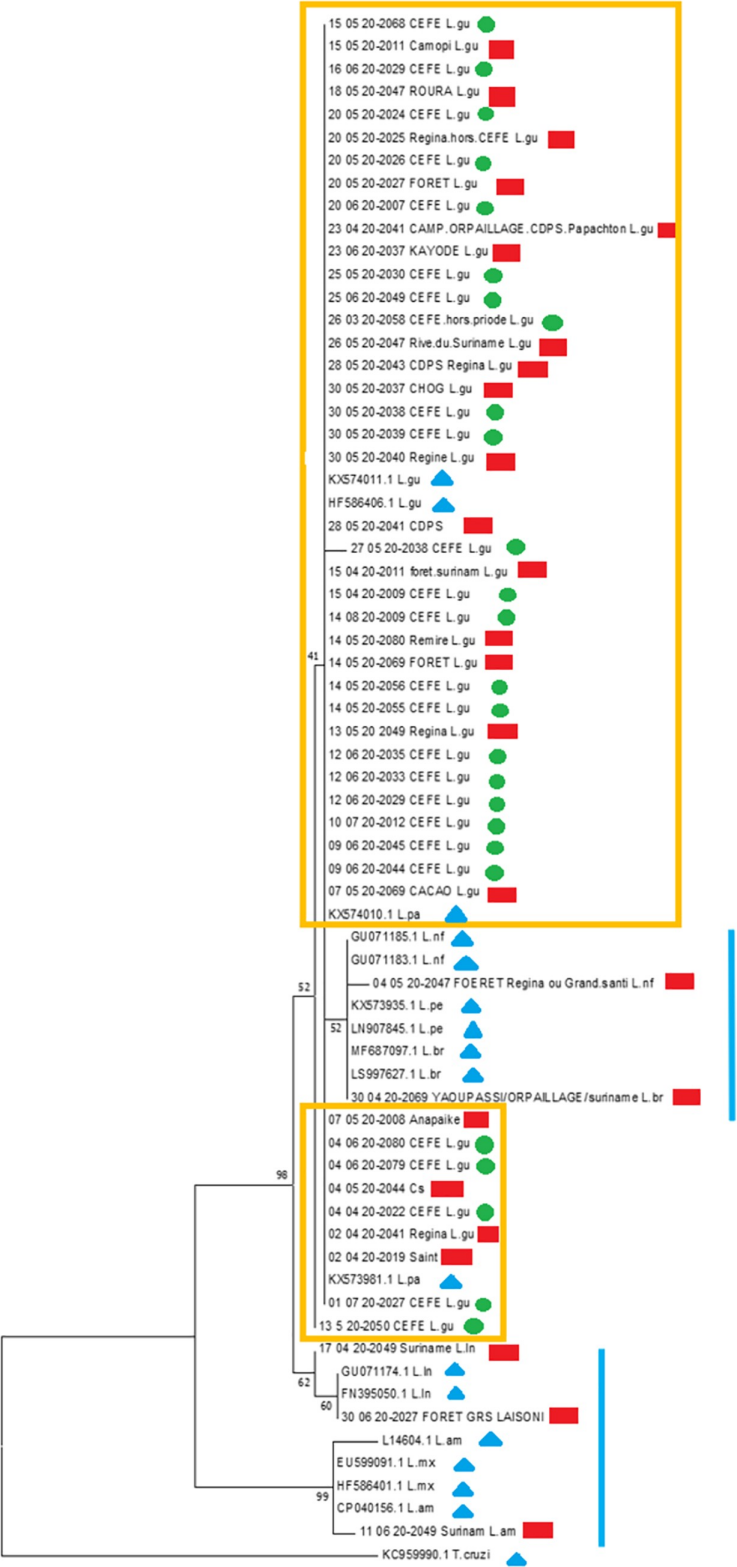
Phylogenetic tree of isolated strains and samples used for comparison, CEFE outbreak, French Guiana, 2020; green dots indicate the 26 strains isolated during the outbreak; red squares indicate 25 other strains isolated in French Guiana during the same period; blue triangles indicate 15 references strains used for species identification.

Concerning the phylogenetic analysis, no specific cluster was identified for the whole set of strains isolated during this outbreak (**Fig 5**). In total, 26 patients had a positive PCR yielding *L*. *guyanensis* and suitable for phylogenetic analysis. These strains are presented in **Fig 5**. Two pseudo-clusters are visible and framed in orange, gathering all the 26 outbreak strains (green dots) but also several samples from civilian patients gathered during the same period throughout French Guiana (red squares). These civilian samples included patients infected in the commune of Regina where the CEFE is located (20-05-20 2025, 30-05-20 2040, 13-05-20 2049, 02-04-20 2041) but also many strains from distant areas such as the Maroni (23-06-20 2037, 07-05-20 2008) or Oyapock (15-05-20 2011) rivers. These pseudo-clusters were, however, well differentiated from reference strains belonging to other species such as *L*. *braziliensis*, *L*. *peruviana*, *L*. *naiffi* or *L*. *lainsoni*, which are underlined in blue on the right side of the tree. Only two samples of *L*. *panamensis* (KX574010.1 KX573981.1*)*, known to be genetically very close to *L*. *guyanensis*, were included in the orange pseudo-clusters, along with two reference strains of *L*. *guyanensis* (KX574011.1 and HF584406.1).

### Epidemiological study

Among the thirty confirmed CL cases of the outbreak, eight could not be reached after their clinical follow-up and were not included in the case-control study (**Fig 1**). In order to recruit controls, we recovered randomly and anonymously a total of 80 phone numbers of CEFE trainees during the same study period who did not present any sign of Cutaneous Leishmaniasis. Among them, 58 were excluded (**Fig 1**). A total of 22 controls were included. The results of this case-control study are presented in **Table 2**.

**Table 2:** Risk factors of Cutaneous Leishmaniasis, case-control study, univariate analysis, confirmed cases of the CEFE outbreak and military controls, French Guiana, 2020.

	Cases	Controls	OR (95%CI)	p-value
**General data**				
**Age (years)**				
• <30	9 (40.9%)	10 (45.45%)	0.83 (0.22–3.22)	0.761
• ≥30	13 (59.1%)	12 (54.6%)	-	
**Rank**				
• Private	19 (86.4%)	13 (59.1%)	4.38 (0.84–29.03)	0.088
• Non-commissioned officer	2 (3.9%)	6 (27.3%)	-	
• Officer	1 (4.6%)	3 (13.6%)		
**Military unit**				
• A	13 (59.1%)	19 (86.36%)	0.24 (0.03–1.18)	0.088
• B	9 (40.9%)	3 (13.64%)	-	
**First time in CEFE**				
• Yes	10 (45.45%)	6 (27.3%)	2.2 (0.54–9.56)	0.210
• No	12 (54.6%)	16 (72.7%)	-	
**Type of training**				
• Fighting	16 (72.7%)	22 (100%)	**0.1 (0–0.74)**	**0.021**
• Rainforest specialist	2 (9.1%)	0	-	
• Jaguar	2 (9.1%)	0		
• No training: instructor	2 (9.1%)	0		
**CEFE period**				
• Week 4 to Week 12	2 (9.1%)	0	-	
• Week 6 to Week 7	1 (4.5%)	9 (40.9%)		
• **Week 13 to Week 14**	**15 (68.2%)**	**7 (31.8%)**	**4.59 (1.1–19.83)**	**0.016**
• Week 16 to Week 17	3 (13.6%)	4 (18.2%)	-	
• Week 19 to Week 20	1 (4.5%)	0		
• Week 21 to Week 22	0	2 (9.1%)		
**Additional survival exercise**				
• Yes	5 (22.7%)	0	5.95 (0.58–305.73)	0.185
• No	17 (77.3%)	22 (100%)	-	
**Knowledge of the disease**				
**Knows that leishmaniasis is transmitted by a sandfly**				
• Yes	21 (95.45%)	19 (86.4%)	3.23 (0.24–182.19)	0.607
• No	1 (4.55%)	3 (13.6%)	-	
**Knows the most at-risk hours of the day**				
• Yes	19 (86.4%)	17 (77.3%)	1.84 (0.30–13.64)	0.698
• No	3 (13.6%)	5 (22.7%)	-	
**Knows that lights attract sandflies**				
• Yes	21 (95.5%)	21 (95.5%)	1 (0.01–82.18)	1
• No	1 (4.5%)	1 (4.5%)	-	
**Can mention three means of prevention against leishmaniasis**				
• Yes	18 (81.8%)	21 (95.5%)	3.32 (0.24–182.18)	0.345
• No	4 (18.2%)	1 (4.5%)	-	
**Behavior during training**				
**Wearing long clothes**				
• Daily	18 (81.8%)	19 (86.4%)	0.72 (0.092–4.89)	1.000
• Sometimes	4 (18.2%)	3 (13.6%)	-	
**Type of hammock used**				
• Provided by the army	15 (68.2%)	11 (50%)	2.14 (0.54–8.78)	0.220
• Personal	7 (31.8%)	11 (50%)	-	
**Use of mosquito net**				
• Yes	22 (100%)	22 (100%)	-	
• No	0	0	-	
**Daily use of mesh hammock**				
• Yes	3 (13.6%)	2 (9.1%)	1.58 (0.16–20.65)	1.000
• No	19 (86.4%))	20 (90.9%)	-	
**Clothes impregnation by soldiers**				
• Yes	3 (13.6%)	2 (9.1%)	1.58 (0.16–20.65)	1.000
• No	19 (86.4%)	20 (90.9%)	-	-
**Skin repellent, frequency of use**				
• Daily	13 (59.1%)	16 (72.7%)	-	
• Sometimes	8 (36.4%)	3 (13.6%)	1.18 (0–2.59)	0.163

Mosquito nets provided to trainees systematically benefited from long-lasting impregnation. Clothes were also pre-impregnated but this impregnation became less effective after several washings and under the damp tropical conditions of forest trainings. Soldiers were advised to regularly perform a new impregnation with an appropriate repellent, but few of them followed this advice (three in the cases group and two in the control group). However the impregnation of clothes was not a significant protective factor. Soldiers undertaking only the “Fighting” course had a lower risk of infection compared with those undertaking longer courses such as “Jaguar” or “Rainforest specialist” or instructors permanently stationed in the CEFE (OR = 0.1 [0–0.74]; p = 0.021). No other individual behaviour was statistically associated with a higher risk of CL. Undertaking a course during week 13 or 14 was associated with a higher risk of infection (OR = 4.59; [1.1–19.83]; p = 0.0159).

During the study period 2285 military personnel were deployed in French Guiana. Among them, 858 took part in the CEFE (**Table 3**). Taking part in a CEFE course was significantly associated with a higher risk of CL infection (OR 2.29 [1.28–4.13], **p = 0.0023**). The attack rate among the 105 members of unit B undertaking a course was significantly higher (OR = 4,22 [1.84–9.53], **p = 0.0001**) than in Regiment A (whose 507 members all took part in one of the CEFE courses) (**Table 3**).

**Table 3:** Comparison of attack rates of CL in French Guiana during the study period, according to attendance of a CEFE course and regiment, 2020.

	Cases of CL	Total	Attack rate	OR (95%IC)	p-value
**Attack rates with or without CEFE training**					
• French Guiana, CEFE excluded	22	1427	1.54%	-	
• CEFE	30	858	3.5%	**OR 2.29 (1.28–4.13)**	**0.0023**
**Attack rates according to regiment**					
• Military unit A	16	507	3.16%	-	
• Military unit B	14	105	13.33%	**OR 4.22 (1.84–9.53)**	**0.0001**

### Environmental investigation

**Fig 6:**
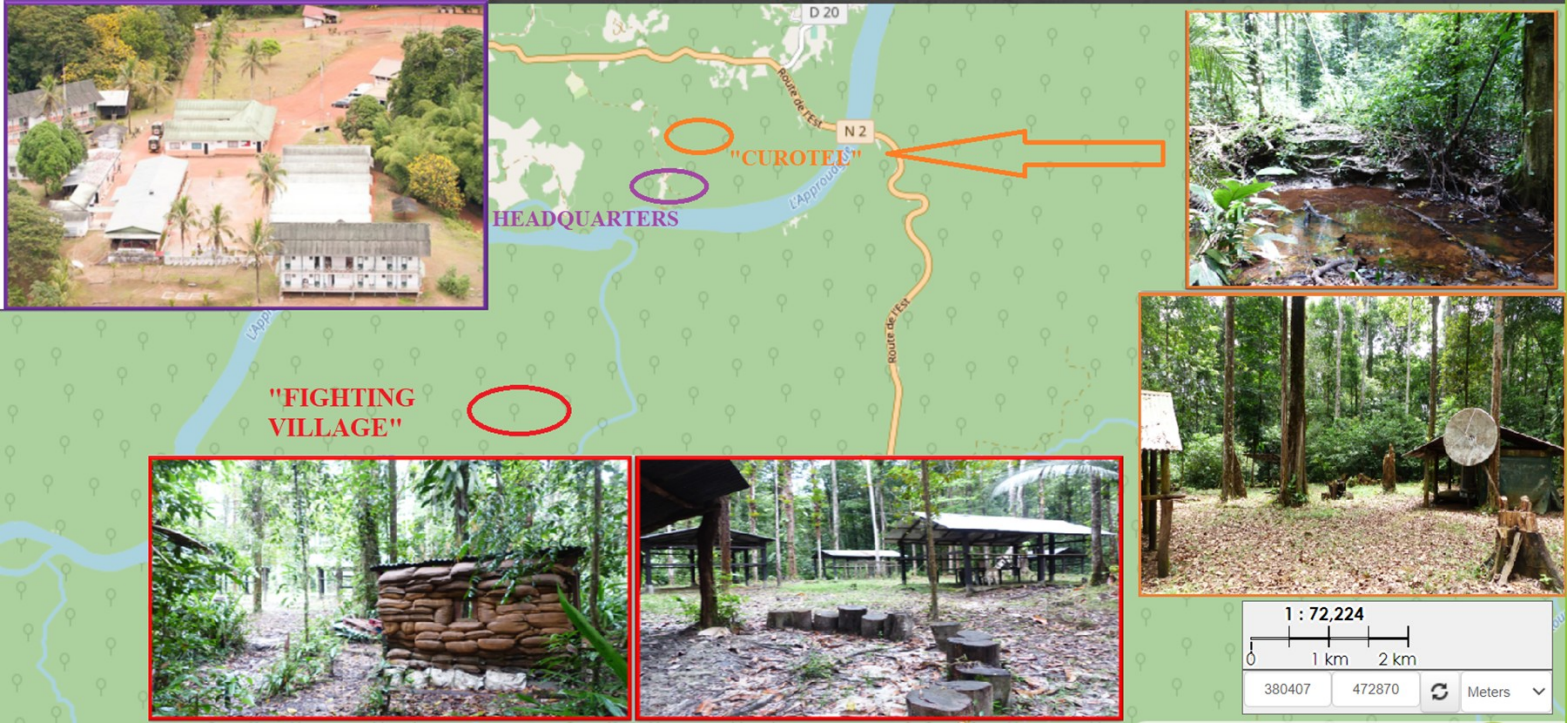
Map of Training Center in Equatorial Forest, CEFE outbreak, French Guiana, 2020, layer from a Guiana Amazonian Park (Parc Amazonien de Guyane) map available at http://cartotheque.parc-amazonien-guyane.fr/index.php/view/map/?repository=pag&project=Limites_PAG.

The whole CEFE area extends over 60km^2^, between the Approuague and Mataroni rivers (**Fig 6**).

Concerning awareness of CL, we performed interviews with officers responsible for the training courses. It appeared that all trainees received information and prevention advices on several tropical diseases, including CL, on the first day of every course. Pieces of advice included protection against small, non-visible mosquitoes; wearing long sleeves and using repellent; holding headlamps with the hand and not wearing it on the forehead. A slight misconception was noted as a focus was given to a local tree named “Bois-cathédrale” (*Chimarrhis turbinate*). This tree was considered by officers as the main shelter for sandflies and soldiers were advised to avoid it but were also falsely reassured if no *C*. *turbinate* was spotted.

Concerning the military gear provided to trainees, it included a Brazilian-model hammock with tight mosquito net and a repellent containing 4% of permethrin. This repellent is normally used for clothing protection but most soldiers misused it and put it on their skin.

Concerning the different exercises performed by the trainees, we looked for activities that could put vectors in contact with humans. Night activities had been stopped in 2016, but started again in 2017. Many exercises were performed close to rivers or streams.

During our tour of the 6000-hectares site, we inspected all zones of encampment or bivouac. Most of them were cleaned and dry areas with only a few well-trimmed trees (**Fig 6**). Only two sites came to our attention as bearing specific high risk for contact with sandflies. The first one was a fighting place nicknamed “Fighting Village” (*Village Combat*) (**Fig 6**). This decoy village is located on the right bank of the Mataroni, about 300 meters from a legal gold mine of 40 hectares. Civilian forest work on the outskirts of this mine were reported by officers and could have increased the population of sandflies in the surroundings of the village. Different platoons are charged with the defence or attack of the position. Service members had to keep a watch during whole nights, sometimes laying on the ground or creeping in the underbrush.

The second high-risk site was nicknamed “Curotel” (**Fig 6**) and formed the ending stage of every training. It is located about 500 meters from headquarters, a few meters away from a small stream (**Fig 6**). Three deforestation places were spotted less than 300 meters from Curotel: an açaï palm farm in expansion; a traditional corn field on slash-and-burn ground; an illegal timber extraction site were dozen of fallen tree trunks were observed.

Concerning the weather during the end of 2019, November appeared to be drier than other years with -52% of rainfall in Regina (compared to normal between 1981 and 2010) [16], followed by a wetter December (+22%) [17] and a very dry January (-67%) [18]. March 2020 was described as the hottest one since 1955, with a mean temperature of 31,7°C and a 32% decrease in rainfall in Regina [19]. These climatic conditions with sharp variations correspond to the highest risk for contaminations [20].

## Discussion

This study explores four aspects of a CL outbreak (clinical, phylogenetic, epidemiological and environmental) in order to explore all possible factors explaining these grouped cases.

Regarding the clinical data, the features observed in this outbreak, such as predominance of *L*. *guyanensis*, ulcerative lesions (90%) or absence of mucosal infection, are in line with usual features observed in French Guiana [3]. However, lesions were more frequently located on the face and the upper limbs (notably the hands), while lower limbs are usually more involved in civilians of French Guiana [3]. This difference is probably explained by the use of uniforms with longs pants. Despite similar species and clinical presentations, ten patients presented a bad outcome, compared to the other 20. Three patients were particularly difficult to heal and needed at least four different schemes. This poor therapeutic response may question the possible resistance of the parasite to the usual treatment by pentamidine. In vitro sensitivity to pentamidine has been studied and identified on promastigote culture in French Guiana, with a strong correlation with patient outcome [21].

Therapeutic failure can also be caused by host factors. Th1 immune response is necessary for the control of *Leishmania* infection [22] but an exacerbation can induce severe CL disease [23]. Th1 response is crucial in the initiation of protection while Th2 response allows parasites to survive by downregulating the Th1 way [24]. Therefore, these failures could be explained by a non-adapted host response to infection.

The presence of RNA viruses in *Leishmania* parasites has been correlated with disease severity and could be an explanation for the occurrence of so many symptomatic cases in a limited period of time [25]. High level of LRV-1 (*Leishmania* RNA Virus 1) has been linked to highly metastasizing *L*. *guyanensis* strains and consequent mucosal clinical presentation [26]. The presence of LRV-1 is also associated with increased intra-lesional inflammatory markers, linked to a first-line treatment failure and symptomatic relapse [27]. But this effect is controversial, as in a more recent study neither the presence nor the genotype of LRV-1 in *L*. *guyanensis* lesions was correlated to treatment failure with pentamidine [28]. Therefore, a systematic search of LRV was not carried out in this outbreak study.

The efficiency of pentamidine as a treatment of CL is not perfect, as shown in Brazil [29]. However, it is usually close to 90% on *L*. *guyanensis* strains observed in French Guiana, as previously published [30]. It is uncommon in this territory to see several patients with treatment failures after pentamidine and amphotericin B, let alone after three different schemes. The efficiency of amphotericin B in CL is a matter of debate and varies importantly between the different published studies [14,31,32]. However, it is the first time that so many treatment schemes are met with failure in French Guiana. This kind of clinical failures should be closely monitored in the future.Five patients were unfortunately lost to follow-up after one month, however this proportion (16.7%) is usually much higher in civilian patients treated in French Guiana.

Regarding the phylogenetic study, we looked for a clonal strain of *Leishmania* which could be responsible for these grouped cases. However, no such clonal infection was observed. Similar results were observed during the small *L*. *braziliensis* outbreak in Saül in 2013 with 5 distinct and non-clustered genotypes [13]. We used the Hsp70 as a target gene for sequencing, as this gene is deemed the best marker for New World CL phylogenetics [33] and is routinely used for species identification in French Guiana [9]. RNA Pol II was once used as a target for routine diagnosis of CL in French Guiana but replaced with Hsp 70 in February 2020. Due to the emergency conditions of this study and the logistical hardships imposed by the Covid-19 pandemics during the study period, we were not able to use other techniques than the routine Hsp70 PCR. Indeed, though Hsp70 allowed us to highlight intra-species differences and geographical clusters for *Leishmania lainsoni* and *naiffi* in a previous study, [5] this gene is known to be highly conserved and is primarily used for differentiation between *Leishmania* species and not as an intra-species marker. Therefore, we could not find correlations between genetic characteristics and clinical or geographical features. Microsatellite markers and/or whole-genome sequencing should be made available for prospective use in future outbreaks. This diversity in isolated strains could also result from the large diversity of species of vectors and reservoirs which can be observed in French Guiana [34,35]. *Ny*. *umbratilis* is the main vector for *L*. *guyanensis* in this region and is usually a canopy-feeding sandfly [36]. However, a study performed along the Brazilian border showed the presence of *Ny*. *umbratilis* carrying *L*. *guyanensis* both on the forest floor and the canopy. It increased the number of possible hosts [37], usually more abundant in the canopy than at the ground level [38]. An important diversity of blood sources for *Ny*. *umbratilis* have been found in this area, such as birds, dogs, armadillo, opossum and humans [34]. Beside *Ny*. *umbratilis*, other phlebotomine species have been associated with *L*. *guyanensis* in French Guiana and neighbouring Brazilian localities: *Psathyromyia dendrophyla* [39], *Ny*. *whitmani*, *Ny*. *anduzei* [35], *Bichromomyia flaviscutellata* [40], *Evandromyia infraspinosa* [34]. These data highlight the possibility that more reservoirs and vectors are involved in CL transmission than originally presumed.

Indeed, concerning the hosts of *L*. *guyanensis*, *C*. *didactylus* is known as the main reservoir [8], while the anteater *Tamandua tetradactyla* and marsupials or rodents play a smaller role [41]. Other mammals such as dogs or monkeys could also be involved [42,43]. Very few trainees reported an encounter or a close contact with animals in CEFE. Therefore, the role played by traditional hosts such as the two-toed sloth seems minimal in this outbreak. *L*. *guyanensis* has also been found in a *Rhipicephalus microplus* tick isolated from a peccary [44]. Interestingly, peccaries were the most frequent animal seen by soldiers in our study. Capture and identification of vectors, measurement of infection rates and identification of infecting species should be prospectively conducted during future training courses to demonstrate the involvement of multiple vectors in infections occurring on the CEFE grounds.

The case-control study did not incriminate any specific individual behavior, as in another military outbreak in 2003 [12]. This could reflect some social desirability bias (military culture is associated with exemplarity and discipline) and a trend to hide non-compliance with prevention measures during interviews. During this 2020 outbreak, we showed that taking part in a CEFE course was associated with a significant higher attack rate than other missions in French Guiana. Therefore, though all service members take part in risky activities such as fighting illegal gold miners in the forest (so-called “Harpie” operations), these activities appear less likely to provide CL infections than a CEFE training. This important risk can be explained by nocturne activities, intense trainings with close contacts with sandflies in fixed stations, compared to longer but itinerant and diurnal missions in the Harpie operations. Being part of unit B was also associated with a significant higher attack rate, which could reflect a better compliance with prevention measures in unit A. Taking part in the “Fighting” course and not in harder courses such as “Jaguar” or “Rainforest specialist” was a significant protective factor, which can be explained by the length and hardships of these exercises. However, the “Survival” exercise, which comes as an extra after ending another course and includes nights in mesh hammock and isolation in the remotest areas of the forest was not associated with infection.

During the environmental investigation, we highlighted two zones which seemed linked with most contaminations: the “Fighting village” and the “Curotel” rest area (**Fig 6**). These areas were surrounded by areas of illegal logging. The flight range of sandflies is around 300 meters, which corresponds to the risky perimeter of CL around deforestation activities [45]. Deforestation appears to be the main trigger for increased CL cases in South America [46]. Human activities play an even more important part than climate in shaping the risk of CL occurrence throughout the different ecosystems of the continent [47]. Extensive literature has described how logging puts humans in contact with sylvatic sandflies, particularly in the absence of local wild reservoirs [46,48,49]. Therefore, a good prevention mean would be to implement a ban on logging in the outskirts of the military area. A geospatial treatment would have provided a better analysis, but soldiers were unable to determine the precise spots on which they were contaminated. We assumed that the favorable conditions of the “Fighting Village” and “Curotel” were the most likely places of contamination but this did not allow us to perform a proper geospatial treatment.

Regarding the information provided to trainees before CEFE courses, a misconception was noted concerning the local tree named “Bois-cathédrale” (*Chimarrhis turbinate*) which was deemed as the main shelter for sandflies, despite the absence of supporting data. This often led the soldiers to disregard other shelters such as creeks or other trees.

Temporal and climatic risk factors are also important. As highlighted in the literature [4,10], the end of the long dry season and the whole period of the short dry season (March) are the more likely periods of infection. In our study, undertaking a CEFE course during the 13^th^ calendar week was associated with a higher risk of infection (OR = 4.59 [1.1–19.83]; **p = 0.0159**). The nocturnal activity of sandflies [45] make night and dusk exercises particularly dangerous. These activities should be avoided during these annual risky periods of climatic shifts from hot and dry to rainy weather. Hot and dry years of El Niño Southern Oscillation are known to yield high number of CL infections and should be feared as risky periods for such trainings [50,51].

When looking for other grouped cases of CL in the literature, a similar investigation can be found concerning an outbreak among military personnel in Peru in 2010 [52]. Very interestingly, many of the findings mirror those of our study: two specific spots in the large training area were incriminated, as well as deforestation and recent land changes. As in our study, the authors raised the hypothesis of multiple exposures and bites but could not confirm it due to the absence of vector captures [52]. The attack rate was much higher (25%) but very few prevention measures against sandfly bites were used before the outbreak (long clothes were not always used and soldiers slept in open rooms). A smaller outbreak (12 cases) was investigated in 2003 in French soldiers infected in French Guiana after taking part in the CEFE and other missions [12]. Military exercises in the forest in a period of high transmission risk was found as a significant risk factor, as in our study. A young age was also incriminated, which was not reported in our findings, though the experience of tropical forest appeared associated with a lower attack rate. As in our study, vector control measures were not statistically significant, maybe due to a small sample size. Conversely, the non-use of repellents and wearing short-sleeves clothes were incriminated as significant risk factors of CL infection among soldiers in Sri Lanka. However, 5000 individuals were screened for this study which was not an outbreak investigation but a large prospective cross-sectional study, which explains how statistical significance could be reached [53]. Among US military forces in Irak, the establishment of a sandfly surveillance program confirmed the presence of high density of infected sandflies on military bases [54]. A vector control program provided less convincing results as insecticides spraying failed to significantly decrease the sandflies populations. Personal protective measures were initially insufficient and consequently targeted by a specific education program. In a military outbreak in the jungle of Panama in 1984, the attack rate was particularly high after a specific exercise on a mortar firing site while other spots were less at-risk [55]. Prevention measures such as repellents were poorly implemented and did not offer efficient protection.

Military outbreaks of CL are associated with very different risk factors than civilian ones. For example in an 2018 study in Yemen, risk factors of infection (agricultural activities of women, malnutrition, proximity of plantations and animals) were mostly linked to the poor living conditions and rural habits of the studied community [56]. A civilian outbreak in the Communidad Valenciana (Spain) led to the implementation of several vector control measures and personal protection against sandfly bites. Dogs were suspected to be the main reservoir of the outbreak but without solid evidence. The only risk factors involved seemed to outdoor activities such as hunting, or the presence of dogs or landfills [57]. Large civilian outbreaks have also been reported in displaced populations, therefore associated with very different causes [58]. Thousands of suspected cases were also reported in Ghana, in an area until then deemed as non-endemic [59]. However, the triggers of this outbreak remain unclear, as in many Sub-Saharan countries where the dynamics of CL infections are poorly studied.

This study has several limitations. As a retrospective study, it can involve a memory bias as military personnel could forget events occurring during the training course. Besides, the small number of cases and controls might explain the absence of significant association between infection and individual behaviors. However, one should bear in mind that large case-controls studies are hard to perform in emergency situations. Moreover, as cases and controls were phoned simultaneously, any memory bias in the case-control study should be evenly shared. Concerning biological tests, immunological studies on biopsies and resistance tests could provide useful data for future investigations. Whole genome sequencing or microsatellite markers would have been very useful. However, neither of microsatellite markers nor whole sequencing are routinely used in the Cayenne Hospital Centre and the implementation of new molecular biology protocols during the pandemics was not achievable. On the other hand Hsp70 was used as a routine marker in samples from all patients but did not allow us to look for geographical or clinical correlations. Whole genome sequencing would have required skin biopsies, which we did not possess for all soldiers, modifying the ethical scope of the study. Sandflies captures were first contemplated on the CEFE site. However, as this investigation was performed several months after the outbreak, and as the long dry season had begun, these data would not have been relevant. However, prospective collections of phlebotomine samples could be contemplated during future CEFE courses.

## Conclusion

This study presents a transdisciplinary approach of a CL outbreak in French Guiana. This investigation highlights the combined risks posed by night exercises, illegal logging and military trainings during at-risk months. Insufficient knowledge of cutaneous leishmaniasis and prevention means might play a role, though no individual behaviour was specifically incriminated. Military training should be adapted to at-risk areas and months of the year. From the clinical point of view, the presence of pentamidine-resistant strains of *L*. *guyanensis* represents a therapeutic challenge which should be closely monitored.
